# Myelopreservation with Trilaciclib in recurrent advanced ovarian cancer: a case report

**DOI:** 10.3389/fonc.2024.1343239

**Published:** 2024-04-22

**Authors:** Huaming Tan, Xiuchen Han, Chao Li, Wenli Liu, Kanghong Li, Xiugui Sheng, Shuying Qi

**Affiliations:** ^1^ Medical College, Shantou University Medical College, Shantou, China; ^2^ National Cancer Center/National Clinical Research Center for Cancer/Cancer Hospital, Shenzhen Hospital, Chinese Academy of Medical Sciences and Peking Union Medical College, Shenzhen, China; ^3^ Cancer Center, Huazhong University of Science and Technology Union Shenzhen Hospital, Shenzhen, China; ^4^ Medical College, Shenzhen University, Shenzhen, China; ^5^ Gynecology Department, Longgang District Maternity and Child Healthcare Hospital of Shenzhen City (Longgang Maternity and Child Institute of Shantou University Medical College), Shenzhen, China; ^6^ Department of Reproductive Medicine, Longgang Central Hospital of Shenzhen, Shenzhen, China

**Keywords:** ovarian cancer, biochemical recurrence, CIM, Trilaciclib, case report

## Abstract

Ovarian cancer is a prevalent malignant tumor of the female reproductive system, often remaining concealed until it reaches an advanced stage. The standard treatment protocol includes cytoreductive surgery for ovarian cancer plus postoperative consolidation chemotherapy and maintenance therapy, although it carries a high recurrence rate. During the treatment period, chemotherapy can lead to bone marrow suppression, a condition known as Chemotherapy-Induced Myelosuppression (CIM). This suppression may necessitate dose reduction or chemotherapy treatment cycle delay. In severe cases, CIM can result in infection, fever, and potential harm to the patient’s life. Here, we report a case of a female patient with ovarian malignant tumor of biochemical recurrence who treated with chemotherapy combined with Trilaciclib, following previous perioperative chemotherapy with occurrence of severe CIM. It involves an intravenous injection of Trilaciclib before chemotherapy, which significantly abates the side effects of chemotherapy, reduces the occurrence of severe CIM, improves the patients’ quality of life, and decreases the economic burden of hospitalization. We hope that this retrospective analysis of the case may serve as a reference in preventing and treating severe CIM during chemotherapy in some patients with malignant tumors, ultimately benefiting more patients with tumors.

## 1 Background

Ovarian cancer is a highly pathogenic reproductive system disease that affects women’s lives. Early diagnosis and treatment can prolong the survival time of patients. Unfortunately, due to the lack of effective screening methods, most women with ovarian cancer are diagnosed at an advanced stage, making the disease very challenging to treat. Ovarian cancer is a highly chemo-sensitive malignancy, and NCCN guidelines recommend platinum-based combination chemotherapy as the first-line chemotherapy regimen for ovarian cancer (1). Although most patients respond to first-line therapy, disease relapse rates are very high and treatment after relapse is more difficulty (2, 3). Hence, monitoring early indicators of tumor relapse, such as human epididymis protein 4 (HE4) or carbohydrate antigen 125 (CA125), as well as more effective chemotherapy regimens remains the primary endpoint for improving OC prognosis (4). However, chemotherapy often leads to multilineage myelosuppression or severe complications that may lead to dose reduction or modification of the chemotherapy regimen. Current treatments for myelosuppression involve stimulating bone marrow hematopoiesis following CIM (5), which only benefit on a single lineage of blood cells or have a potential risk of bone marrow depletion. For instance, recombinant human granulocyte colony-stimulating factor (rhG-CSF) and PEGylated -rhG-CSF (PEG-rhG-CSF) are primarily used to address decreases in WBC and ANC caused by chemo-radiotherapy. Trilaciclib (Cosela™) is a small molecule, short-acting cyclin-dependent kinase (CDK) 4 and 6 inhibitor developed by G1 Therapeutics (6). It has shown myeloprotective properties and potential antitumor efficacy and safety in combination with cancer chemotherapy. CDKs regulate cell cycle progression, and Trilaciclib induces transient, reversible G1 cell cycle arrest of proliferating hematopoietic stem and progenitor cells in the bone marrow. This mechanism protects these cells from damage during chemotherapy, reducing the incidence of CIM in cancer patients. Trilaciclib has demonstrated efficacy in reducing the occurrence of CIM and the need for supportive care interventions, all without compromising the clinical benefit of the chemotherapy regimen during treatment with acceptable safety (7). This article presents a case study illustrating the bone marrow protective effects and chemotherapy efficacy of Trilaciclib in patients with ovarian malignancies.

## 2 Case data

A 68-year-old female patient presented with a height of 152 cm and a weight of 39 kg. On March 1, 2023, the patient was diagnosed with a biochemical recurrence of an ovarian malignant tumor after postoperative chemotherapy, confirmed by tumor marker detection and CT imaging.

### 2.1 Past history

On July, 2018, the patient began experiencing diarrhea along with abdominal pain.

On October 15, 2018, tumor markers carbohydrate antigen (CA125) > 800 U/mL, and human epididymis protein 4 (HE4) 192 pmol/L. Abdominal DR revealed intestinal obstruction, and chest and abdominal CT revealed a mass lesion at the rectosigmoid junction, multiple lymph node metastases in the hilar region and retroperitoneum, omental metastases, liver metastases, pelvic and abdominal effusion, and pseudomyxoma peritonei. A colonoscopic biopsy revealed that the mass at the rectosigmoid junction was moderately differentiated adenocarcinoma.

From 2018-11-26 to 2019-01-09, the patient underwent 3 cycles of neoadjuvant therapy (paclitaxel liposome + carboplatin). During this period, the patient experienced severe bone marrow suppression on several occasions, which improved with symptomatic treatment (see **Table 1** for detailed treatment information).

**Table 1:** Chemotherapy regimens and management of CIM.

Time	Cycle	Treatment Regimen	CIM	G-CSF	TPO	IL-11	Other
Neoadjuvant chemotherapy							
2018-11-26	1	Paclitaxel liposome 90 mg d1, d8 + carboplatin 0.2 g d1, d8	III °	75 μg x 14			
2018-12-18	2	Paclitaxel liposome 90 mg d1, d8 + carboplatin 0.2 g d1, d8	III °	75 μg x 14			L/C/X/A/H
2019-01-09	3	Paclitaxel liposome 90 mg d1, d8 + carboplatin 0.2 g d1, d8	III °	75 μg x 16		3 mg × 7	L/C/X/A/H
Surgery							
2019-02-11		Surgical treatment in Department of Gynecology, Cancer Hospital Shenzhen, Chinese Academy of Medical Sciences	III °	100 mg x 2	15000U x 3		Y/L/C/A/H
Adjuvant therapy							
2019-03-22	1	Paclitaxel liposome 90 mg d1, d8 + carboplatin 0.2 g d1, d8	IV ° ^a^	75 μg x 26			
2019-04-13	2	Paclitaxel liposome 60 mg d1, d8 + carboplatin 0.2 g d1, d8	III °	75 μg x 18			Y/A/H L/C/X/A/H
2019-05-06	3	Paclitaxel liposome 60 mg d1, d8 + carboplatin 0.2 g d1, d8	III °	75 μg x 26	15000U x 6	3 mg x 9	L/C/X/W/A/H
2019-06-03	4	Paclitaxel liposome 60 mg d1, d8 + nedaplatin 0. 0 5 g d1, d8	III °	75 μg x 16			L/C/X/Z
2019-07-01		None	IV °	75 μg x 10	15000U x 8	3 mg x 5	
2019-10-15		None	I °				C/X/W/Z
Systemic therapy ^b^							
2023-03-07~ 2023-06-14	1- 15	Paclitaxel liposome 60 mg d1 + carboplatin 0.1 g d1 + bevacizumab 0.1 g d1	I - II °^b^				H
2023-06-20	16	Paclitaxel liposome 60 mg d1 + carboplatin 0.1 g d1 + bevacizumab 0.1 g d1	I °	75 μg x 4			H
2023-06-28	17	Paclitaxel liposome 60 mg d1 + carboplatin 0.1 g d1 + bevacizumab 0.1 g d1	I °	75 μg x 4	15000U x 4		H
2023-07-06	18	Paclitaxel liposome 60 mg d1 + carboplatin 0.1 g d1 + bevacizumab 0.1 g d1	II °	75 μg x 4			H

a: concomitant febrile neutropenia.

b: From 2023-3-7 to 2023-7-6 for 18 treatment weeks, using G - CSF treatment (75 μg x 4) vials only at Weeks 16, 17 and 18. Treated with TPO (15000U x 4) only after Week 17. Intravenous injection of Trilaciclib 300 mg within 4 h before chemotherapy for the whole course of weekly treatment; liver protection/stomach protection/antiemetic therapy for the whole course without other cytoprotectants, traditional Chinese medicine or other drugs.

Y: Amino acid fat emulsion intravenous nutrition; L: Leucogen; W: Ubenimex; A: Amifostine; X: β-elemene; C: Shenqi Fuzheng; Z: traditional Chinese medicine; H: liver protection/stomach protection/antiemetic.

On February 11, 2019, patient underwent total abdominal adnexectomy + omentectomy + appendectomy + pelvic lymphadenectomy + para-aortic lymphadenectomy + partial proctectomy + intestinal anastomosis + abdominopelvic adhesiolysis. After 5 cycles of adjuvant paclitaxel liposome + carboplatin therapy, severe myelosuppression occurred several times during the operation and was improved after symptomatic treatment (see **Table 1** for detailed treatment). Throughout the entire treatment period, the patient experienced severe fatigue and anorexia, and needed symptomatic and supportive treatment with Amifostine, amino acid fat emulsion for intravenous nutrition, Chinese herbs, etc. Regular check-ups were conducted thereafter.

Smoking history: No smoking history.

ECOG score: 2.

### 2.2 Diagnosis

On February 27, 2023, reexamination of CA125 showed a level of 177.00 U/mL. The CT report showed no obvious mass, but there is consideration of biochemical recurrence.

2023-03-01: CA125 181.00 U/ml, carcinoembryonic antigen (CEA) 1.24 ng/mL, and HE 4 61.3 pmol/L [CA125 and HE4 were determined by an electrochemiluminescence immunoassay (Roche Diagnostics GmbH)].

A gynecological examination revealed a negative vulva, smooth vagina, smooth stump, no palpable mass in the pelvic cavity, and a slightly thickened area palpable at the obturator fossa of the left pelvic wall.

Urinary color ultrasound revealed the following: 1. Bilateral kidney and bladder sonography showed no significant abnormalities. 2. Bilateral ureters showed no significant dilatation.

Transabdominal gynecological color Doppler examination indicated: 1. Sonographic changes after total hysterectomy. 2. No obvious abnormal echoes in both adnexa.

Hepatobiliary, pancreatic and splenic color ultrasound results: 1. Intrahepatic cystic space-occupying lesion, considering the possibility of a hepatic cyst. 2. The sonograms of the gallbladder, spleen and pancreas showed no significant abnormalities.

Blood cell analysis: WBC: 3.3*10^9^/L, absolute neutrophil count: 2.34*10^9^/L, RBC: 4.56*10^12^/L, hemoglobin: 125 g/L, Platelet count 220*10^9^/L. Diagnosis Ovarian malignancy.

### 2.3 Treatment

2023-03-07, 2023-03-14, 2023-03-21, 2023-03-28, 2023-04-04, 2023-04-11, 2023-04-19, 2023-04-26, 2023-05-03, 2023-05-10, 2023-05-18, 2023-05-26, 2023-06-01, 2023-06-07, 2023-06-14, 2023-06-20, 2023-06-28, 2023-07-06, 18 weeks of chemotherapy: paclitaxel liposome 60 mg d1 + carboplatin 0.1 g d1 + bevacizumab Monoclonal antibody 0.1 g d1.

Adjuvant drug: Trilaciclib 300 mg via intravenous infusion for 30 min d1.

### 2.4 Treatment outcome

**Figure 1:**
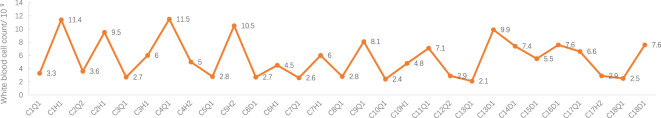
White blood cell count during treatment. C: week of treatment; Q: before treatment; H: after treatment; D: before chemotherapy on the day of treatment.

The patient had previously experienced severe CIM in this regimen, which was extremely challenging to treat due to significant side effects of chemotherapy and the high cost of treatment and adverse reaction management. As a result, a new treatment regimen was developed, involving paclitaxel liposome + carboplatin + bevacizumab administered weekly for 18 weeks. During the treatment, the side effects of chemotherapy were closely monitored. Additionally, prophylactic administration of Trilaciclib with chemotherapy was administered to safeguard the patient’s bone marrow. It is well-known that CDK4/6 is a critical regulator of the transition from G1 to S phase of the cell cycle (8), while Trilaciclib is a highly selective CDK4/6 inhibitor that arrests the cell cycle of bone marrow hematopoietic stem cells in G1 phase, shielding them from the cytotoxic effects of chemotherapeutic agents, thereby reducing adverse effects such as bone marrow suppression caused by chemotherapy (9). Throughout the entire treatment, the patient maintained good physical condition with no noticeable chemotherapy-related side effects. A blood routine reexamination on the second day after the completion of chemotherapy indicated an increase in white blood cells (see **Figure 1** for details), leading to the patient’s discharge. Outpatient blood routine showed no significant abnormality.

**Figure 2:**
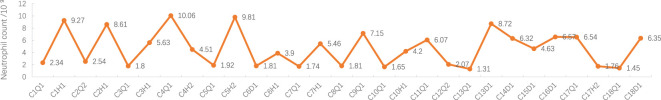
Neutrophil count during treatment. C: week of treatment; Q: before treatment; H: after treatment; D: before chemotherapy on the day of treatment.

**Figure 3:**
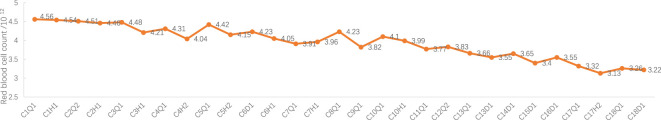
Red blood cell count during treatment. C: week of treatment; Q: before treatment; H: after treatment; D: before chemotherapy on the day of treatment.

**Figure 4:**
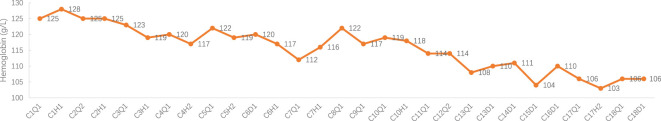
Hemoglobin Concentration during treatment. C: week of treatment; Q: before treatment; H: after treatment; D: before chemotherapy on the day of treatment.

**Figure 5:**
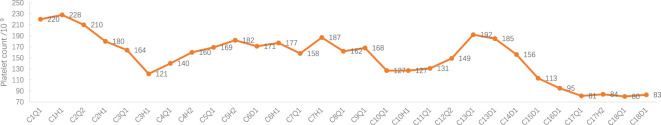
Platelet count during treatment. C: week of treatment; Q: before treatment; H: after treatment; D: before chemotherapy on the day of treatment.

**Figure 6:**
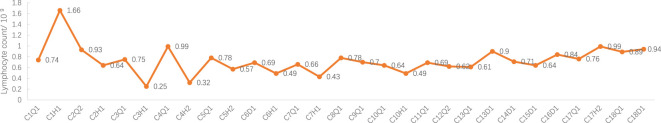
Lymphocyte count during treatment. C: week of treatment; Q: before treatment; H: after treatment; D: before chemotherapy on the day of treatment.

Adverse events (AEs): Myelosuppression grade 1-2 (mild, see **Figures 1**–**6**), no other non-hematological adverse events.

CIM medication use: Symptomatic treatment with G-CSF at Week 16-18 and thrombopoietin (TPO) at Week 18 (**Table 1**)

**Figure 7:**
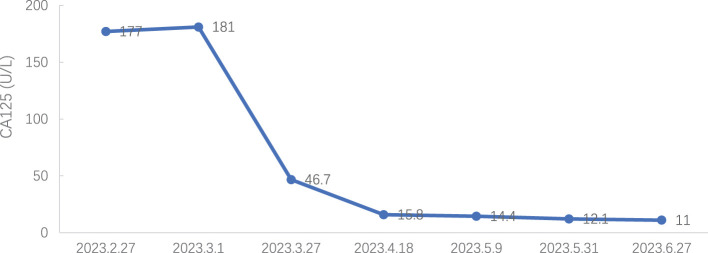
Carbohydrate antigen (CA125) expression during treatment.

**Figure 8:**
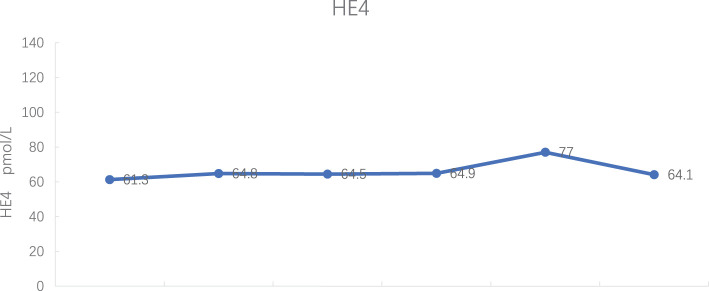
Human epididymis protein 4 (HE4) expression during treatment.

Tumor markers: CA 125 decreased to normal levels (see **Figure 7**), and HE 4 expression was within normal levels (see **Figure 8**).

Quality of life: No influence on daily life behavior.

Efficacy evaluation: No delay in chemotherapy cycle, no reduction in chemotherapy dose.

Radiographic evaluation: No radiographic progression.

## 3 Discussion

Currently, CA125 is a biomarker used to detect ovarian cancer recurrence, monitor response to treatment and changes in chemotherapeutic strategy (10). In addition, the FDA approved HE4 and CA125 for OC monitoring or prognostic markers (4). Hence, it is very necessary for postoperative detection of CA125 and HE4 in patients with ovarian cancer.

Chemotherapy plays a crucial role in the treatment of ovarian cancer, especially for recurrent ovarian cancer, and CIM resulting from standard chemotherapy significantly impacts its efficacy. A meta-analysis revealed that the most common adverse reactions observed during chemotherapy treatment include grade 2 alopecia, grade 3-4 neutropenia, grade 3 neurotoxicity, thrombocytopenia, mucositis, hand-foot syndrome, and nausea (11). Moreover, the combination of carboplatin and paclitaxel exhibited an incidence of grade 3/4 neutropenia at 27.5% and grade 3/4 thrombocytopenia at 3.2% (12). Paclitaxel dose-dense regimens have demonstrated the extension of PFS and OS in ovarian cancer patients but have also led to increased overall toxicity (13). Therefore, CIM management is necessary for patients with ovarian cancer. Clinically, G-CSF, TPO drugs, enhanced intravenous nutrition therapy, and adjuvant therapy with traditional Chinese medicine are usually used for such patients. However, some patients still experience moderate to severe myelosuppression under these treatment measures, necessitating the reduction of chemotherapeutic drug doses, modification of the chemotherapy regimen, or delay in chemotherapy timing (13, 14). Additionally, due to adverse reactions to chemotherapy, patients endure prolonged hospital stays, intravenous drug infusion, and inherent resistance to treatment, all of which affect the efficacy of chemotherapy and ultimately lead to tumor resistance and/or disease progression. Previous drugs for CIM treatment, such as G-CSF, TPO, erythropoiesis-stimulating agents (ESA), stimulate the proliferation and differentiation of bone marrow hematopoietic stem cells into myeloid cells. However, these cytokines can only target specific lineages of myeloid cells. Treating CIM requires multiple cell-stimulating factors, leading to a slow onset of action, a long treatment cycle, and high economic medication costs (15). Moreover, repeated mobilization of bone marrow hematopoietic stem cells with multiple doses of cell-stimulating factors can lead to bone marrow depletion, thereby limiting the total duration of chemotherapy in patients (16, 17).

Trilaciclib is a highly potent, selective, and reversible CDK4/6 inhibitor that induces temporary arrest of bone marrow hematopoietic stem/progenitor cells (HSPCs) in the G1 phase. It alleviates the depletion of HSPCs by reducing the burden of replication of HSPCs through transient G1 arrest (18). Trilaciclib used before chemotherapy can transiently arrest HSPCs in G1 phase, thereby shielding them from the damage caused by chemotherapeutic drugs and preserving bone marrow hematopoietic function. Unlike traditional treatments, Trilaciclib fully safeguards bone marrow hematopoiesis and does not lead to bone marrow depletion from the source.

In this case, intravenous injection of Trilaciclib before chemotherapy significantly improved the side effects of chemotherapy, enhanced the quality of life for patients, and reduced the hospitalization expenses. Regarding myelosuppression, three years ago, the patient experienced severe grade 3/4 myelosuppression and granulocytopenia with fever during anti-tumor therapy, including the perioperative period. Chemotherapy drugs led to only grade 2 myelosuppression with anterior intravenous injection of Trilaciclib, both during treatment and the post-treatment phase. Regarding the patient’s quality of life, the patient suffered severe fatigue, anorexia and other discomfort during chemotherapy 3 years ago and required intravenous nutrition therapy. With the Trilaciclib treatment, the patient experienced improved self-feeling, was free from obvious discomfort, maintained a regular diet and night sleep, experienced less alopecia, and retained a normal lifestyle. Interestingly, we found that this patient had an increase in all three blood routine tests on the second day after chemotherapy when white blood cells were lower than 3.0 x 10^9^/L and neutrophils were > 1.5 x 10^9^/L. Do these phenomena suggest that we could lower the minimum standard of the three lines before chemotherapy? Furthermore, the cost of CIM treatment significantly decreased and length of hospital stay reduced. Besides, HE4 expression levels were within the normal range during the treatment period, and CA125 expression decreased to the upper limit of normal from 7^th^ cycle, indicating that Trilaciclib did not antagonize the efficacy of chemotherapy for patients with biochemical relapse.

A comparison between the initial treatment and retreatment after relapse, Trilaciclib effectively protected bone marrow stem cells during chemotherapy, reduced the degree of bone marrow suppression, minimized the side effects of chemotherapy, enhanced the compliance of patients with chemotherapy, and ensured that the treatment was performed on schedule. Additionally, it reduced the length of hospital stay, the hospitalization costs, and the reliance on colony-stimulating factors such as G-CSF, TPO, ESA, et al. However, this drug is currently expensive and has low patient accessibility. In combination, the price of Trilaciclib can offset and/or reduce the cost of leukocyte-elevating or platelet-elevating treatment, hospitalization costs, and examination costs required by patients due to myelosuppression during chemotherapy, ultimately reducing the overall cost of treatment for patients. In addition, this case suggests that Trilaciclib may be potential myelopreservation benefit in other tumors, where the current standard therapy relies on highly myelotoxic chemotherapy or chemosensitive tumors, such as etoposide combined with carboplatin regimen for small cell lung cancer, platinum- or paclitaxel-containing regimen for triple-negative breast cancer, platinum-based regimen for non-small cell lung cancer, and osteosarcoma with high-intensity chemotherapy which may cause severe CIM.

In summary, during chemotherapy for patients with malignant tumors, Trilaciclib can effectively protect normal human cells, reduce the side effects of chemotherapy, especially reduce bone marrow suppression, enhance patient chemotherapy compliance, ensure on-schedule treatment, shorten the length of hospital stay, reduce hospitalization costs, decrease the use of related drugs, and ultimately ensure that anti-tumor chemotherapy is performed as scheduled to achieve a good therapeutic effect. This greatly enhances patient satisfaction whether for grade I prevention or grade II prevention in CIM.

Based on the efficacy and mechanism of action of Trilaciclib, the following points can be further explored in clinical applications:

1. Determine whether the corresponding target can be genetically detected to screen the significantly beneficial population, non-beneficial population or tumor progression population. For example, Trilaciclib could protect cancer stem cells in some cancers resulting in poor response to chemotherapy.
2. Trilaciclib can promote increased levels of chemokines CXCL9 and CXCL10, up-regulate the expression of PD-L1 protein in tumor cells, and facilitate the infiltration of effector T cells into tumors. It also up-regulates MHC I/II on the surface of cancer cells and increases the expression of IFN gene, enhancing the antigen presentation of tumor cells. Additionally, it enhances T cell function by protecting bone marrow hematopoietic stem cells. These results suggest that Trilaciclib may activate the anticancer activity of immune cells (19). Therefore, it is worth exploring whether it can be used in combination with immunosuppressive agents to enhance the efficacy of anti-tumor immunotherapy.
3. For patients receiving cell therapy, investigate whether Trilaciclib be infused before the infusion of autoimmune cells to temporarily inhibit immune cell proliferation, reduce autoimmune reactions, and increase anti-tumor efficacy.
4. Trilaciclib was found to reduce DNA damage induced by multiple chemotherapeutic agents in normal cells by inducing transient G1 arrest in a dose-dependent manner (9). Since DNA damage increases the risk of developing tumors, this suggests that Trilaciclib has the potential to prevent the development of other tumors due to chemotherapeutic agents.

## Data availability statement

The original contributions presented in the study are included in the article/supplementary material. Further inquiries can be directed to the corresponding authors.

## Ethics statement

Written informed consent was obtained from the individual(s) for the publication of any potentially identifiable images or data included in this article.

## Author contributions

HT: Writing – original draft. XH: Data curation, Writing – review & editing. CL: Data curation, Writing – review & editing. WL: Visualization, Writing – review & editing. KL: Visualization, Writing – review & editing. XS: Writing – review & editing. SQ: Writing – review & editing.
